# Personal

**Published:** 1905-12

**Authors:** 


### PERSONAL.

Dr. Franklin C. Gram, who for the past 14 years was located at 460 Glenwood Av., has removed to the commodious office and home which he recently erected at 849 Humboldt Parkway.

---

Dr. Albert Goldspohn, of Chicago, a Fellow of the American Association of Obstetricians and Gynecologists, has given $25, 000 to build a science hall at the Northwest College, Naperville, Illinois.

---

Dr. Lewis C. Morris, of Birmingham, was elected president of the Jefferson County (Alabama) Medical Society at the annual meeting held December 18, 1905.

---

Dr. Brooks H. Wells, of New York, editor of the American Journal of Obstetrics, has removed from 34 West 45th street to 523 Madison avenue. Hours, 11:30 to 1, and by appointment. Telephone 2566 Plaza.

---

Dr. Roland O. Meisenbach, formerly of Saint Louis, announces his entry into private practice at 140 Allen Streret, Buffalo. Practice limited to orthopedics. Dr. Miesenbach has served as Orthopedic House Surgeon, Carney Hospital, Orthopedic House Surgeon, at the House of the Good Samaritan, and Orthopedic Assistant at the Children's Hospital, Boston.

---

Dr. Joseph Price, of Philadelphia, recently paid a visit to Dallas, Texas, on the invitation of the Dallas County Medical Society. He spent two days as the guest of the society, made ten operations, and delivered an address lasting an hour and three-quarters, besides several clinical lectures. He also participated in breakfasts, luncheons, dinners and receptions, the hospitality of Dallas being unlimited. An automobile was provided for his rapid transit from point to point, thus economising time. It was a great ovation to one of the greatest American surgeons. It was a strenuous forty-eight hours; few men could have withstood it. But Dr. Price proved equal to the demands upon him —physically, surgically and intellectually,—and returned home rested and rejuvenated by his little outing.

---

Dr. Smith Ely Jelliffe, of New York, editor of the Medical News until January 1, 1906, has joined the editorial corps of the New York Medical Journal. Dr. Jelliffe's experience as a medical editor will make him a valuable member of Dr. Foster's staff.

---

Dr. Lewis C. Bosher, of Richmond, Va., has acquired the valuable property No. 422 East Franklin Street in that city, to which he has removed his residence and offices. Hours 9-11, 4-5. Sundays, 9-11 only. Telephone 160.

---

Dr. Joseph Roby, deputy health officer of Rochester, read an important paper before the New York State Dairymen's Convention, recently held at Binghamton. He chose for his subject "Economy in Producing and Marketing Certified Milk."

